# Supplementary material for: Comparative Transcriptome Analysis Provided a New Insight into the Molecular Mechanisms of Epididymis Regulating Semen Volume in Drakes
Source: Animals (Basel). 2022 Nov 3;12(21):3023. doi: 10.3390/ani12213023 (PMC9655896; doi:10.3390/ani12213023)
Supplement: Supplementary file 1 [file animals-12-03023-s001.zip › Supplementary Figure S1.pdf]

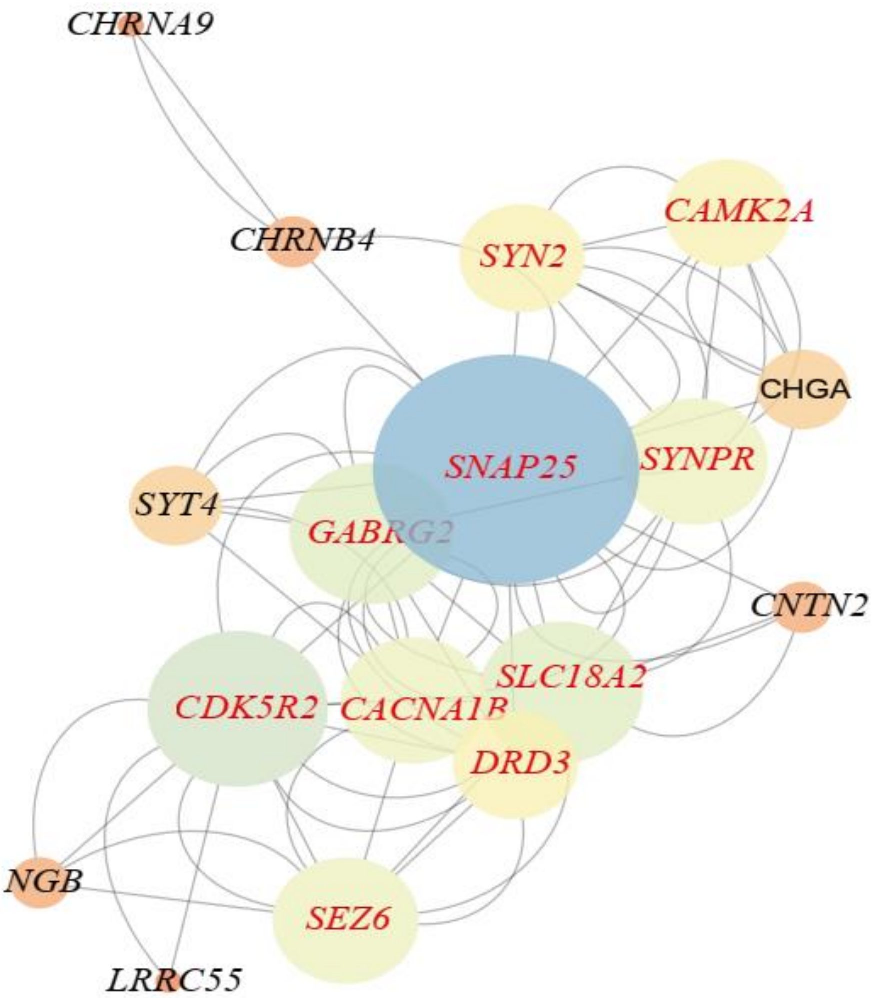

Supplementary Figure S1. PPI enrichment analysis showing physical interactions formed among DEGs. The number of nodes of genes highlighted in red was more than ten.
