# Supplementary material for: Comparative Transcriptome Analysis Provided a New Insight into the Molecular Mechanisms of Epididymis Regulating Semen Volume in Drakes
Source: Animals (Basel). 2022 Nov 3;12(21):3023. doi: 10.3390/ani12213023 (PMC9655896; doi:10.3390/ani12213023)
Supplement: Supplementary file 1 [file animals-12-03023-s001.zip › Supplementary Table S1.pdf]

Supplementary Table S1. Sequence comparison results of RNA-seq data with the reference genomes of duck.

Sample	Raw Reads	Clean Reads	Q20 (%)	Q30 (%)	GC Content (%)	Mapping Rate (%)
HSV1	51932972	51514192	98.59%	95.71%	50.30%	90.38%
HSV2	52566378	52102974	98.50%	95.48%	50.19%	90.31%
HSV3	52538370	52063874	98.49%	95.44%	50.38%	91.00%
LSV1	51764764	51336562	98.55%	95.59%	50.47%	91.24%
LSV2	50705946	50303856	98.52%	95.50%	49.75%	91.16%
LSV3	51531425	51028092	98.56%	95.65%	50.97%	91.07%
