# Supplementary material for: Comparative Transcriptome Analysis Provided a New Insight into the Molecular Mechanisms of Epididymis Regulating Semen Volume in Drakes
Source: Animals (Basel). 2022 Nov 3;12(21):3023. doi: 10.3390/ani12213023 (PMC9655896; doi:10.3390/ani12213023)
Supplement: Supplementary file 1 [file animals-12-03023-s001.zip › Supplementary Table S2.pdf]

Supplementary Table S2. Gene ontological analysis results of DEGs that were identified from the epididymis of drakes with different semen volume.

P value	Rich_factor	Gene onthology	DEGs
		**Biological process**	
Hormone secretion			
4.52E-05	0.038	positive regulation of cortisol secretion	*CRH, GAL*
5.56E-05	0.077	response to estrogen	*CRH, GAL, PTPRN, STAR*
7.53E-05	0.038	cortisol secretion	*CRH, GAL*
7.53E-05	0.038	regulation of cortisol secretion	*CRH, GAL*
7.53E-05	0.038	positive regulation of glucocorticoid secretion	*CRH, GAL*
1.57E-04	0.038	positive regulation of corticosteroid hormone secretion	*CRH, GAL*
2.03E-04	0.096	insulin secretion	*CHGA, CRH, GAL, PTPRN, SNAP25*
2.69E-04	0.038	positive regulation of steroid hormone secretion	*CRH, GAL*
3.36E-04	0.038	regulation of glucocorticoid secretion	*CRH, GAL*
3.40E-04	0.096	hormone metabolic process	*ACE2, GAL, NR5A1, PRLHR, STAR*
4.89E-04	0.096	peptide hormone secretion	*CHGA, CRH, GAL, PTPRN, SNAP25*
4.91E-04	0.038	glucocorticoid secretion	*CRH, GAL*
8.86E-04	0.038	regulation of corticosteroid hormone secretion	*CRH, GAL*
1.13E-03	0.038	corticosteroid hormone secretion	*CRH, GAL*
1.26E-03	0.038	regulation of steroid hormone secretion	*CRH, GAL*

1.33E-03	0.096	hormone secretion	*CHGA, CRH, GAL, PTPRN, SNAP25*
1.56E-03	0.096	hormone transport	*CHGA, CRH, GAL, PTPRN, SNAP25*
1.69E-03	0.038	glucocorticoid metabolic process	*GAL, STAR*
1.84E-03	0.038	steroid hormone secretion	*CRH, GAL*
2.70E-03	0.058	regulation of steroid metabolic process	*GAL, NR5A1, STAR*
4.49E-03	0.038	response to mineralocorticoid	*CRH, STAR*
5.01E-03	0.077	regulation of hormone secretion	*CHGA, CRH, GAL, SNAP25*
5.55E-03	0.019	positive regulation of corticosterone secretion	*CRH*
5.72E-03	0.096	response to peptide hormone	*CAMK2A, GAL, NR5A1, PTPRN, STAR*
6.35E-03	0.038	negative regulation of peptide hormone secretion	*CHGA, CRH*
8.19E-03	0.038	endocrine hormone secretion	*CRH, GAL*
8.31E-03	0.019	brain renin-angiotensin system	*ACE2*
8.31E-03	0.019	negative regulation of luteinizing hormone secretion	*CRH*
8.31E-03	0.019	cellular response to luteinizing hormone stimulus	*STAR*
Neurotransmitter synthesis, transport			
5.27E-09	0.173	neurotransmitter transport	*CADPS, CAMK2A, CHRNB4, CSPG5, DRD3, SLC18A2, SNAP25, SYN2, SYT4*
6.49E-09	0.173	regulation of neurotransmitter levels	*CADPS, CAMK2A, CHRNB4, CSPG5, DRD3, SLC18A2, SNAP25, SYN2, SYT4*
1.44E-07	0.135	neurotransmitter secretion	*CADPS, CAMK2A, CHRNB4, CSPG5, SNAP25, SYN2, SYT4*
3.14E-07	0.115	amine transport	*ACE2, CHGA, CRH, DRD3, SLC18A2, SYT4*
1.77E-06	0.096	catecholamine transport	*CHGA, CRH, DRD3, SLC18A2, SYT4*
3.55E-06	0.096	monoamine transport	*CHGA, CRH, DRD3, SLC18A2, SYT4*

5.27E-06	0.096	regulation of amine transport	*ACE2, CHGA, CRH, DRD3, SYT4*
5.27E-06	0.154	exocytosis	*CADPS, CDK5R2, CHGA, CSPG5, RAB3C, SNAP25, SYN2, SYT4*
7.20E-06	0.058	negative regulation of catecholamine secretion	*CHGA, CRH, SYT4*
8.37E-06	0.096	regulation of neurotransmitter transport	*CAMK2A, CHRNB4, CSPG5, DRD3, SYT4*
1.28E-05	0.135	organic hydroxy compound transport	*CHGA, CRH, DRD3, GAL, SLC18A2, STAR, SYT4*
1.75E-05	0.077	regulation of catecholamine secretion	*CHGA, CRH, DRD3, SYT4*
2.01E-05	0.077	catecholamine secretion	*CHGA, CRH, DRD3, SYT4*
5.64E-05	0.058	negative regulation of amine transport	*CHGA, CRH, SYT4*
9.49E-05	0.077	regulation of neurotransmitter secretion	*CAMK2A, CHRNB4, CSPG5, SYT4*
2.31E-04	0.058	neurotransmitter uptake	*DRD3, SLC18A2, SNAP25*
3.61E-04	0.058	dopamine transport	*DRD3, SLC18A2, SYT4*
4.09E-04	0.038	response to histamine	*DRD3, GABRG2*
5.48E-04	0.096	peptide secretion	*CHGA, CRH, GAL, PTPRN, SNAP25*
1.39E-03	0.038	serotonin transport	*CRH, SLC18A2*
1.46E-03	0.096	amide transport	*CHGA, CRH, GAL, PTPRN, SNAP25*
1.69E-03	0.038	response to corticosterone	*CRH, STAR*
1.84E-03	0.038	neurotransmitter receptor internalization	*DRD3, SNAP25*
2.78E-03	0.019	7,8-dihydroneopterin 3'-triphosphate biosynthetic process	*GCH1*
2.78E-03	0.019	sequestering of neurotransmitter	*SLC18A2*
3.19E-03	0.058	neuropeptide signaling pathway	*GAL, PRLHR, SSTR1*
4.01E-03	0.058	response to alkaloid	*CRH, DRD3, STAR*
4.25E-03	0.038	neurotransmitter reuptake	*DRD3, SLC18A2*
4.49E-03	0.038	dopamine secretion	*DRD3, SYT4*
4.49E-03	0.038	regulation of dopamine secretion	*DRD3, SYT4*

5.25E-03	0.038	dopamine metabolic process	*DRD3, GCH1*
Transmembrane signaling			
1.50E-10	0.25	modulation of chemical synaptic transmission	*CACNA1B, CAMK2A, CHRNB4, CNTN2, CRH, CSPG5, DRD3, LRRC4C, NPTXR, SNAP25, STAR, SYT4, TNR*
1.54E-10	0.25	regulation of trans-synaptic signaling	*CACNA1B, CAMK2A, CHRNB4, CNTN2, CRH, CSPG5, DRD3, LRRC4C, NPTXR, SNAP25, STAR, SYT4, TNR*
3.82E-09	0.231	signal release	*CADPS, CAMK2A, CHGA, CHRNB4, CRH, CSPG5, DRD3, GAL, PTPRN, SNAP25, SYN2, SYT4*
1.44E-07	0.135	signal release from synapse	*CADPS, CAMK2A, CHRNB4, CSPG5, SNAP25, SYN2, SYT4*
3.22E-06	0.135	regulated exocytosis	*CADPS, CDK5R2, CHGA, CSPG5, SNAP25, SYN2, SYT4*
1.86E-05	0.115	vesicle-mediated transport in synapse	*CADPS, CSPG5, DRD3, SLC18A2, SNAP25, SYN2*
2.97E-05	0.077	calcium-ion regulated exocytosis	*CADPS, CDK5R2, SYN2, SYT4*
3.46E-05	0.096	regulation of G protein-coupled receptor signaling pathway	*CAMK2A, CHGA, CNTN2, DRD3, MRAP*
5.02E-05	0.058	positive regulation of G protein-coupled receptor signaling pathway	*CHGA, CNTN2, MRAP*
1.44E-04	0.096	synaptic vesicle cycle	*CADPS, CSPG5, SLC18A2, SNAP25, SYN2*
5.66E-04	0.077	regulation of postsynaptic membrane potential	*CHRNA9, CHRNB4, GABRG2, SEZ6*
8.12E-04	0.096	peptide transport	*CHGA, CRH, GAL, PTPRN, SNAP25*
9.30E-04	0.058	negative regulation of synaptic transmission	*DRD3, SYT4, TNR*
9.75E-04	0.096	positive regulation of ion transport	*ACE2, CAMK2A, CRH, GAL, LRRC55*
1.13E-03	0.038	positive regulation of calcium ion-dependent exocytosis	*CDK5R2, SYT4*
1.76E-03	0.058	positive regulation of exocytosis	*CADPS, CDK5R2, SYT4*
2.31E-03	0.115	regulation of ion transmembrane transport	*ACE2, CACNA1B, CRH, DRD3, GAL, LRRC55*

2.36E-03	0.038	synaptic transmission, dopaminergic	*CRH, DRD3*
2.36E-03	0.038	adenylate cyclase-activating adrenergic receptor signaling pathway	*CHGA, DRD3*
2.54E-03	0.038	positive regulation of potassium ion transmembrane transporter activity	*GAL, LRRC55*
2.78E-03	0	vesicle fusion with vesicle	*SYT4*
2.78E-03	0.019	adenylate cyclase-activating adrenergic receptor signaling pathway involved in cardiac muscle relaxation	*CHGA*
2.78E-03	0.019	negative regulation of dense core granule exocytosis	*SYT4*
3.06E-03	0.096	regulation of cation transmembrane transport	*ACE2, CRH, DRD3, GAL, LRRC55*
3.10E-03	0.058	synaptic vesicle exocytosis	*CADPS, CSPG5, SNAP25*
3.19E-03	0.058	chemical synaptic transmission, postsynaptic	*CHRNA9, CHRNB4, SEZ6*
3.45E-03	0.058	regulation of potassium ion transport	*DRD3, GAL, LRRC55*
3.56E-03	0.038	adrenergic receptor signaling pathway	*CHGA, DRD3*
4.47E-03	0.077	potassium ion transport	*DRD3, GAL, LRRC55, SNAP25*
4.73E-03	0.038	vesicle docking involved in exocytosis	*RAB3C, SNAP25*
4.74E-03	0.077	anion transmembrane transport	*ACE2, GABRG2, SLC13A1, SLC35D3*
5.25E-03	0.038	regulation of calcium ion-dependent exocytosis	*CDK5R2, SYT4*
5.27E-03	0.096	regulation of metal ion transport	*CAMK2A, CRH, DRD3, GAL, LRRC55*
5.55E-03	0.019	regulation of proline import across plasma membrane	*ACE2*
5.55E-03	0.019	positive regulation of proline import across plasma membrane	*ACE2*

5.55E-03	0.019	regulation of L-proline import across plasma membrane	*ACE2*
5.55E-03	0.019	positive regulation of L-proline import across plasma membrane	*ACE2*
6.19E-03	0.096	calcium ion transport	*CACNA1B, CAMK2A, CHRNA9, CRH, DRD3*
6.35E-03	0.038	calcium-dependent cell-cell adhesion via plasma membrane cell adhesion molecules	*CDH7, CDHR3*
6.35E-03	0.038	lipid export from cell	*CRH, GAL*
6.35E-03	0.038	positive regulation of potassium ion transmembrane transport	*GAL, LRRC55*
7.77E-03	0.058	adenylate cyclase-activating G protein-coupled receptor signaling pathway	*CHGA, DRD3, MRAP*
8.31E-03	0.019	UDP-glucuronic acid transmembrane transport	*SLC35D3*
8.31E-03	0.019	UDP-N-acetylgalactosamine transmembrane transport	*SLC35D3*
8.31E-03	0.019	clustering of voltage-gated potassium channels	*CNTN2*
8.31E-03	0.019	synaptic transmission involved in micturition	*CHRNB4*
8.31E-03	0.019	positive regulation of adenosine receptor signaling pathway	*CNTN2*
8.51E-03	0.038	positive regulation of potassium ion transport	*GAL, LRRC55*
8.51E-03	0.058	positive regulation of cation transmembrane transport	*ACE2, GAL, LRRC55*
Transmembrane transporter activity			
1.02E-03	0.096	regulation of transmembrane transporter	*ACE2, CRH, DRD3, GAL, LRRC55*

		activity	
1.35E-03	0.077	regulation of signaling receptor activity	*ACE2, CRH, LYPD1, NPTXR*
1.66E-03	0.096	regulation of transporter activity	*ACE2, CRH, DRD3, GAL, LRRC55*
1.69E-03	0.077	regulation of cation channel activity	*CRH, DRD3, GAL, LRRC55*
5.55E-03	0.019	negative regulation of sodium:proton antiporter activity	*DRD3*
5.55E-03	0.019	positive regulation of large conductance calcium-activated potassium channel activity	*GAL*
6.40E-03	0.077	regulation of ion transmembrane transporter activity	*CRH, DRD3, GAL, LRRC55*
8.31E-03	0.019	regulation of large conductance calcium-activated potassium channel activity	*GAL*
Nervous system development			
1.22E-05	0.096	metencephalon development	*CDK5R2, CEND1, PHOX2A, SEZ6, SSTR1*
1.76E-05	0.115	regulation of synaptic plasticity	*CAMK2A, CNTN2, CRH, SNAP25, STAR, TNR*
2.88E-05	0.154	regulation of neuron projection development	*CNTN2, ITM2C, LRRC4C, SEZ6, SHOX2, SNAP25, TNR, VIM*
6.11E-05	0.096	hindbrain development	*CDK5R2, CEND1, PHOX2A, SEZ6, SSTR1*
7.53E-05	0.038	hindbrain maturation	*CDK5R2, CEND1*
1.57E-04	0.038	central nervous system maturation	*CDK5R2, CEND1*
1.64E-04	0.077	cerebellum development	*CDK5R2, CEND1, SEZ6, SSTR1*
3.36E-04	0.038	pons development	*CDK5R2, PHOX2A*
3.40E-04	0.058	regulation of neuronal synaptic plasticity	*CAMK2A, CNTN2, STAR*
5.05E-04	0.058	telencephalon cell migration	*CDK5R2, CNTN2, TNR*
5.89E-04	0.058	forebrain cell migration	*CDK5R2, CNTN2, TNR*
8.77E-04	0.077	regulation of axonogenesis	*CNTN2, LRRC4C, SHOX2, TNR*
9.20E-04	0.077	neuron migration	*CAMK2A, CDK5R2, CNTN2, TBX20*

1.06E-03	0.077	central nervous system neuron differentiation	*CEND1*, *CNTN2*, *PHOX2A*, *TBX20*
1.46E-03	0.096	gliogenesis	*CDK5R2*, *CNTN2*, *CSPG5*, *DRD3*, *VIM*
2.36E-03	0.038	cerebellar Purkinje cell layer development	*CEND1*, *SEZ6*
2.63E-03	0.058	long-term synaptic potentiation	*CRH*, *SNAP25*, *TNR*
2.70E-03	0.058	excitatory postsynaptic potential	*CHRNA9*, *CHRNB4*, *SEZ6*
2.78E-03	0.019	trochlear nerve morphogenesis	*PHOX2A*
2.78E-03	0.019	trochlear nerve formation	*PHOX2A*
2.78E-03	0.019	cerebellar granular layer maturation	*CEND1*
2.78E-03	0.019	locus ceruleus development	*PHOX2A*
2.78E-03	0.019	radial glia guided migration of cerebellar granule cell	*CEND1*
2.78E-03	0.019	establishment of protein localization to juxtaparanode region of axon	*CNTN2*
2.93E-03	0.038	spinal cord motor neuron differentiation	*PHOX2A*, *TBX20*
3.32E-03	0.096	regulation of neurogenesis	*BRINP1*, *DRD3*, *SHOX2*, *STAR*, *TNR*
3.50E-03	0.077	glial cell differentiation	*CNTN2*, *CSPG5*, *DRD3*, *VIM*
3.94E-03	0.096	forebrain development	*CDK5R2*, *CNTN2*, *CRH*, *SSTR1*, *TNR*
4.11E-03	0.058	glial cell development	*CNTN2*, *CSPG5*, *VIM*
5.55E-03	0.019	trochlear nerve development	*PHOX2A*
5.55E-03	0.019	pons maturation	*CDK5R2*
5.55E-03	0.019	oculomotor nerve morphogenesis	*PHOX2A*
5.55E-03	0.019	oculomotor nerve formation	*PHOX2A*
5.55E-03	0.019	superior olivary nucleus development	*CDK5R2*
5.55E-03	0.019	superior olivary nucleus maturation	*CDK5R2*
5.55E-03	0.019	negative regulation of cerebellar granule cell precursor proliferation	*CEND1*

5.55E-03	0.019	negative regulation of sprouting of injured axon	*TNR*
5.55E-03	0.019	negative regulation of axon extension involved in regeneration	*TNR*
5.55E-03	0.019	glial cell projection elongation	*CSPG5*
6.44E-03	0.096	synapse organization	*CNTN2, GABRG2, LRRC4C, SEZ6, TNR*
6.52E-03	0.058	negative regulation of neuron projection development	*ITM2C, TNR, VIM*
6.64E-03	0.038	ventral spinal cord development	*PHOX2A, TBX20*
6.92E-03	0.058	negative regulation of neurogenesis	*BRINP1, DRD3, TNR*
7.56E-03	0.096	regulation of nervous system development	*BRINP1, DRD3, SHOX2, STAR, TNR*
7.62E-03	0.058	negative regulation of nervous system development	*BRINP1, DRD3, TNR*
8.19E-03	0.038	cell differentiation in spinal cord	*PHOX2A, TBX20*
8.19E-03	0.038	cerebellar cortex development	*CEND1, SEZ6*
8.31E-03	0.019	visceral motor neuron differentiation	*TBX20*
8.31E-03	0.019	oculomotor nerve development	*PHOX2A*
8.31E-03	0.019	cerebellum maturation	*CEND1*
8.31E-03	0.019	cerebellar cortex maturation	*CEND1*
8.31E-03	0.019	lateral mesoderm morphogenesis	*TBX20*
8.31E-03	0.019	lateral mesoderm formation	*TBX20*
8.31E-03	0.019	regulation of sprouting of injured axon	*TNR*
8.31E-03	0.019	regulation of axon extension involved in regeneration	*TNR*
Other			
6.04E-07	0.135	locomotory behavior	*CEND1, CNTN2, DRD3, SEZ6, SLC18A2, SNAP25, TNR*

1.17E-05	0.135	developmental maturation	*CDK5R2, CEND1, CNTN2, GAL, PTPRN, SEZ6, SYT4*
1.24E-05	0.115	regulation of blood pressure	*ACE2, CHGA, CRH, DRD3, GCH1, POSTN*
2.59E-05	0.058	circadian sleep/wake cycle, sleep	*CRH, DRD3, STAR*
2.99E-05	0.096	adult behavior	*CEND1, CNTN2, DRD3, GABRG2, SEZ6*
3.92E-05	0.058	circadian sleep/wake cycle process	*CRH, DRD3, STAR*
4.52E-05	0.038	secretory granule maturation	*PTPRN, SYT4*
4.88E-05	0.096	learning	*CNTN2, CRH, DRD3, SNAP25, TNR*
5.64E-05	0.058	circadian sleep/wake cycle	*CRH, DRD3, STAR*
5.72E-05	0.135	protein secretion	*CHGA, CRH, DRD3, GAL, PTPRN, RAB3C, SNAP25*
5.83E-05	0.135	establishment of protein localization to extracellular region	*CHGA, CRH, DRD3, GAL, PTPRN, RAB3C, SNAP25*
6.48E-05	0.077	associative learning	*CRH, DRD3, SNAP25, TNR*
6.69E-05	0.135	protein localization to extracellular region	*CHGA, CRH, DRD3, GAL, PTPRN, RAB3C, SNAP25*
7.25E-05	0.115	learning or memory	*BRINP1, CNTN2, CRH, DRD3, SNAP25, TNR*
9.47E-05	0.058	sleep	*CRH, DRD3, STAR*
1.13E-04	0.038	circadian sleep/wake cycle, REM sleep	*CRH, STAR*
1.64E-04	0.115	cognition	*BRINP1, CNTN2, CRH, DRD3, SNAP25, TNR*
1.72E-04	0.058	positive regulation of heart contraction	*ACE2, CHGA, GCH1*
2.00E-04	0.058	positive regulation of blood circulation	*ACE2, CHGA, GCH1*
2.11E-04	0.115	positive regulation of secretion	*CADPS, CDK5R2, CRH, DRD3, GAL, SYT4*
2.31E-04	0.058	negative regulation of blood pressure	*CRH, DRD3, GCH1*
2.40E-04	0.096	regulation of exocytosis	*CADPS, CDK5R2, CSPG5, RAB3C, SYT4*
2.69E-04	0.038	positive regulation of cardiac muscle contraction	*ACE2, CHGA*
2.69E-04	0.038	dense core granule exocytosis	*CADPS, SYT4*
3.01E-04	0.058	circadian behavior	*CRH, DRD3, STAR*

3.36E-04	0.038	luteinization	*NR5A1, PTPRN*
3.40E-04	0.058	rhythmic behavior	*CRH, DRD3, STAR*
6.74E-04	0.038	positive regulation of striated muscle contraction	*ACE2, CHGA*
7.37E-04	0.077	negative regulation of secretion by cell	*CHGA, CRH, DRD3, SYT4*
1.01E-03	0.058	adult locomotory behavior	*CEND1, CNTN2, SEZ6*
1.01E-03	0.058	multicellular organismal response to stress	*BRINP1, CRH, GCH1*
1.09E-03	0.096	positive regulation of secretion by cell	*CADPS, CDK5R2, CRH, GAL, SYT4*
1.13E-03	0.038	regulation of circadian sleep/wake cycle, sleep	*CRH, DRD3*
1.29E-03	0.077	negative regulation of secretion	*CHGA, CRH, DRD3, SYT4*
1.39E-03	0.096	rhythmic process	*CRH, DRD3, NR5A1, PTPRN, STAR*
1.56E-03	0.077	negative regulation of cell development	*BRINP1, DRD3, POSTN, TNR*
1.59E-03	0.058	endocrine process	*ACE2, CRH, GAL*
1.69E-03	0.038	regulation of circadian sleep/wake cycle	*CRH, DRD3*
1.76E-03	0.077	negative regulation of cell projection organization	*HRG, ITM2C, TNR, VIM*
1.84E-03	0.038	exploration behavior	*BRINP1, TNR*
2.18E-03	0.038	response to immobilization stress	*CRH, GAL*
2.36E-03	0.038	benzene-containing compound metabolic process	*HAAO, STAR*
2.41E-03	0.058	regulation of systemic arterial blood pressure	*ACE2, CRH, POSTN*
2.41E-03	0.058	regulation of cell morphogenesis involved in differentiation	*CNTN2, CSPG5, POSTN*
2.41E-03	0.058	cellular response to ketone	*CRH, POSTN, STAR*
2.54E-03	0.038	adult walking behavior	*CEND1, CNTN2*

2.55E-03	0.077	regulation of heart contraction	*ACE2, CHGA, GCH1, SHOX2*
2.63E-03	0.058	feeding behavior	*ACE2, GAL, PRLHR*
2.73E-03	0.038	walking behavior	*CEND1, CNTN2*
2.78E-03	0.019	foramen ovale closure	*TBX20*
2.78E-03	0.019	embryonic heart tube elongation	*TBX20*
2.78E-03	0.019	avascular cornea development in camera-type eye	*ANGPTL7*
2.78E-03	0.019	pulmonary vein morphogenesis	*TBX20*
2.78E-03	0.019	negative regulation of vasculature development involved in avascular cornea development in camera-type eye	*ANGPTL7*
2.78E-03	0.058	cellular biogenic amine metabolic process	*DRD3, GCH1, HAAO*
2.93E-03	0.038	cardiac atrium morphogenesis	*SHOX2, TBX20*
2.93E-03	0.038	response to pain	*CRH, GCH1*
2.93E-03	0.038	cellular response to dexamethasone stimulus	*CRH, STAR*
3.54E-03	0.058	phenol-containing compound metabolic process	*DRD3, GCH1, STAR*
3.61E-03	0.077	import into cell	*ACE2, DRD3, SLC18A2, SNAP25*
3.73E-03	0.077	anatomical structure maturation	*CDK5R2, CEND1, CNTN2, GAL*
3.82E-03	0.058	receptor internalization	*CNTN2, DRD3, SNAP25*
3.82E-03	0.058	cellular amine metabolic process	*DRD3, GCH1, HAAO*
4.01E-03	0.038	behavioral fear response	*BRINP1, CRH*
4.25E-03	0.038	behavioral defense response	*BRINP1, CRH*
4.31E-03	0.058	amine metabolic process	*DRD3, GCH1, HAAO*
4.47E-03	0.077	heart contraction	*ACE2, CHGA, GCH1, SHOX2*
4.49E-03	0.038	cardiac atrium development	*SHOX2, TBX20*

4.49E-03	0.038	regulation of endocrine process	*CRH, GAL*
4.73E-03	0.038	cardiac conduction system development	*MSC, SHOX2*
4.73E-03	0.038	secretory granule organization	*PTPRN, SYT4*
4.99E-03	0.038	fear response	*BRINP1, CRH*
5.16E-03	0.077	heart process	*ACE2, CHGA, GCH1, SHOX2*
5.25E-03	0.038	response to dexamethasone	*CRH, STAR*
5.53E-03	0.077	regulation of blood circulation	*ACE2, CHGA, GCH1, SHOX2*
5.55E-03	0.019	angiotensin-mediated drinking behavior	*ACE2*
5.55E-03	0.019	sinoatrial valve development	*SHOX2*
5.55E-03	0.019	pulmonary valve formation	*TBX20*
5.55E-03	0.019	insecticide metabolic process	*STAR*
5.55E-03	0.019	phthalate metabolic process	*STAR*
5.55E-03	0.019	negative regulation of circadian sleep/wake cycle, REM sleep	*CRH*
5.55E-03	0.019	musculoskeletal movement, spinal reflex action	*DRD3*
5.55E-03	0.019	dihydrobiopterin metabolic process	*GCH1*
5.55E-03	0.019	positive regulation of timing of catagen	*GAL*
5.55E-03	0.019	regulation of proline transport	*ACE2*
5.55E-03	0.019	positive regulation of relaxation of cardiac muscle	*CHGA*
5.55E-03	0.019	positive regulation of type B pancreatic cell proliferation	*PTPRN*
5.55E-03	0.019	peptidyl-threonine autophosphorylation	*CAMK2A*
5.55E-03	0.019	positive regulation of dense core granule biogenesis	*CHGA*

6.07E-03	0.038	cellular response to alkaloid	*CRH*, *STAR*
6.07E-03	0.096	cell junction assembly	*ACE2*, *CDH7*, *CDHR3*, *GABRG2*, *HRG*
6.39E-03	0.058	regulation of regulated secretory pathway	*CDK5R2*, *CSPG5*, *SYT4*
6.49E-03	0.077	regulation of protein secretion	*CHGA*, *CRH*, *DRD3*, *SNAP25*
6.64E-03	0.038	negative regulation of peptide secretion	*CHGA*, *CRH*
6.92E-03	0.077	cell-cell adhesion via plasma-membrane adhesion molecules	*CDH7*, *CDHR3*, *CNTN2*, *LRRC4C*
1.01E+00	1.077	cell-cell adhesion via plasma-membrane adhesion molecules	*CDH7*, *CDHR3*, *CNTN2*, *LRRC5C*
6.94E-03	0.038	positive regulation of muscle contraction	*ACE2*, *CHGA*
7.06E-03	0.058	response to interferon-gamma	*GCH1*, *STAR*, *VIM*
7.77E-03	0.058	cell-cell junction assembly	*ACE2*, *CDH7*, *CDHR3*
8.06E-03	0.058	neuromuscular process	*DRD3*, *GCH1*, *TNR*
8.19E-03	0.038	adherens junction organization	*CDH7*, *CDHR3*
8.19E-03	0.038	defense response to fungus	*CHGA*, *HRG*
8.31E-03	0.019	cardiac right atrium morphogenesis	*SHOX2*
8.31E-03	0.019	primary sex determination	*NR5A1*
8.31E-03	0.019	positive regulation of circadian sleep/wake cycle, wakefulness	*CRH*
8.31E-03	0.019	branchiomeric skeletal muscle development	*MSC*
8.31E-03	0.019	biphenyl metabolic process	*STAR*
8.31E-03	0.019	dibenzo-p-dioxin metabolic process	*STAR*
8.31E-03	0.019	protein localization to secretory granule	*CHGA*
8.31E-03	0.019	catagen	*GAL*
8.31E-03	0.019	anthranilate metabolic process	*HAAO*
8.31E-03	0.019	regulation of timing of catagen	*GAL*

8.31E-03	0.019	dense core granule biogenesis	*CHGA*
8.31E-03	0.019	positive regulation of bile acid biosynthetic process	*STAR*
8.31E-03	0.019	positive regulation of relaxation of muscle	*CHGA*
8.31E-03	0.019	positive regulation of bile acid metabolic process	*STAR*
8.31E-03	0.019	dense core granule maturation	*PTPRN*
8.31E-03	0.019	regulation of endocannabinoid signaling pathway	*CAMK2A*
8.31E-03	0.019	negative regulation of female gonad development	*NR5A1*
8.31E-03	0.019	regulation of dense core granule biogenesis	*CHGA*
8.51E-03	0.058	cellular response to glucose stimulus	*CRH*, *PTPRN*, *STAR*
		**Cellular component**	
Transmembrane transporter activity			
1.26E-03	0.089	ion channel complex	*CACNA1B, CHRNA9, CHRNB4, GABRG2, LRRC55*
8.35E-03	0.036	SNARE complex	*SNAP25, SYN2*
1.14E-02	0.018	synaptobrevin 2-SNAP-25-syntaxin-1a-complexin I complex	*SNAP25*
1.42E-02	0.018	calcium- and calmodulin-dependent protein kinase complex	*CAMK2A*
Nervous system development			
4.81E-06	0.054	neuronal dense core vesicle	*CADPS, CHGA, SYT4*
5.07E-06	0.143	glutamatergic synapse	*CADPS, CSPG5, DRD3, LRRC4C, NPTXR, SNAP25, SYN2, TNR*

1.28E-05	0.143	synaptic membrane	*CHRNA9, CHRNB4, CNTN2, CSPG5, DRD3, GABRG2, LRRC4C, SNAP25*
1.36E-05	0.125	postsynaptic membrane	*CHRNA9, CHRNB4, CNTN2, CSPG5, DRD3, GABRG2, LRRC4C*
1.50E-05	0.089	synaptic vesicle membrane	*RAB3C, SLC18A2, SYN2, SYNPR, SYT4*
1.50E-05	0.089	exocytic vesicle membrane	*RAB3C, SLC18A2, SYN2, SYNPR, SYT4*
2.13E-05	0.143	transport vesicle	*CHGA, PTPRN, RAB3C, SLC18A2, SNAP25, SYN2, SYNPR, SYT4*
2.58E-05	0.089	intrinsic component of postsynaptic membrane	*CHRNA9, CNTN2, CSPG5, DRD3, LRRC4C*
2.66E-05	0.107	synaptic vesicle	*RAB3C, SLC18A2, SNAP25, SYN2, SYNPR, SYT4*
3.13E-05	0.107	transport vesicle membrane	*PTPRN, RAB3C, SLC18A2, SYN2, SYNPR, SYT4*
4.26E-05	0.107	exocytic vesicle	*RAB3C, SLC18A2, SNAP25, SYN2, SYNPR, SYT4*
8.01E-05	0.036	neuronal dense core vesicle membrane	*CADPS, SYT4*
1.03E-04	0.089	intrinsic component of synaptic membrane	*CHRNA9, CNTN2, CSPG5, DRD3, LRRC4C*
3.30E-04	0.054	intrinsic component of synaptic vesicle membrane	*RAB3C, SYNPR, SYT4*
3.52E-04	0.071	integral component of postsynaptic membrane	*CHRNA9, CSPG5, DRD3, LRRC4C*
7.12E-04	0.071	neuron projection terminus	*GCH1, PTPRN, SLC18A2, SYT4*
8.97E-04	0.071	integral component of synaptic membrane	*CHRNA9, CSPG5, DRD3, LRRC4C*
1.02E-03	0.054	GABA-ergic synapse	*CSPG5, DRD3, GABRG2*
1.30E-03	0.054	integral component of postsynaptic specialization membrane	*CHRNA9, DRD3, LRRC4C*
1.45E-03	0.054	Schaffer collateral - CA1 synapse	*LRRC4C, SYN2, TNR*
1.45E-03	0.054	intrinsic component of postsynaptic specialization membrane	*CHRNA9, DRD3, LRRC4C*
3.48E-03	0.089	postsynaptic specialization	*CAMK2A, CHRNA9, DRD3, LRRC4C, SYN2*

3.91E-03	0.089	transmembrane transporter complex	*CACNA1B, CHRNA9, CHRNB4, GABRG2, LRRC55*
4.51E-03	0.036	integral component of synaptic vesicle membrane	*SYNPR, SYT4*
4.60E-03	0.054	postsynaptic specialization membrane	*CHRNA9, DRD3, LRRC4C*
5.72E-03	0.018	perineuronal net	*TNR*
5.72E-03	0.018	intrinsic component of neuronal dense core vesicle membrane	*SYT4*
8.35E-03	0.036	integral component of postsynaptic density membrane	*DRD3, LRRC4C*
9.39E-03	0.036	intrinsic component of postsynaptic density membrane	*DRD3, LRRC4C*
9.97E-03	0.071	distal axon	*CDK5R2, PTPRN, SLC18A2, SNAP25*
1.21E-02	0.071	secretory granule membrane	*CADPS, CHRNB4, SNAP25, SYT4*
1.28E-02	0.054	anchored component of membrane	*CNTN2, LYPD1, RAB3C*
1.36E-02	0.054	extrinsic component of plasma membrane	*CDH7, CDHR3, SNAP25*
1.42E-02	0.018	clathrin-sculpted monoamine transport vesicle	*SLC18A2*
1.42E-02	0.018	clathrin-sculpted monoamine transport vesicle membrane	*SLC18A2*
1.42E-02	0.018	anchored component of postsynaptic membrane	*CNTN2*
1.47E-02	0.071	postsynaptic density	*CAMK2A, DRD3, LRRC4C, SYN2*
1.55E-02	0.071	asymmetric synapse	*CAMK2A, DRD3, LRRC4C, SYN2*
1.56E-02	0.071	endocytic vesicle	*ACE2, CAMK2A, DRD3, VIM*
1.63E-02	0.036	main axon	*CNTN2, CRH*
Other			

6.20E-05	0.054	dense core granule	*CADPS, CHGA, SYT4*
1.68E-04	0.036	dense core granule membrane	*CADPS, SYT4*
9.42E-04	0.036	acetylcholine-gated channel complex	*CHRNA9, CHRNB4*
3.55E-03	0.036	catenin complex	*CDH7, CDHR3*
4.63E-03	0.089	transporter complex	*CACNA1B, CHRNA9, CHRNB4, GABRG2, LRRC55*
5.72E-03	0.018	intrinsic component of dense core granule membrane	*SYT4*
7.30E-03	0.089	collagen-containing extracellular matrix	*ANGPTL1, ANGPTL7, HRG, POSTN, TNR*
8.57E-03	0.018	protein kinase 5 complex	*CDK5R2*
8.57E-03	0.018	perisynaptic extracellular matrix	*TNR*
8.57E-03	0.018	synapse-associated extracellular matrix	*TNR*
1.05E-02	0.054	perikaryon	*CRH, NGB, PTPRN*
		**Molecular function**	
Hormone secretion			
3.21E-03	0.017	corticotropin-releasing hormone activity	*CRH*
4.16E-03	0.034	neuropeptide hormone activity	*CRH, GAL*
6.41E-03	0.017	corticotropin hormone receptor binding	*MRAP*
6.41E-03	0.017	type 5 melanocortin receptor binding	MRAP
Transmembrane signaling			
3.21E-03	0.017	3-hydroxyanthranilate 3,4-dioxygenase activity	*HAAO*
4.31E-03	0.102	channel activity	*CACNA1B, CHRNA9, CHRNB4, GABRG2, LRRC55, SNAP25*
4.38E-03	0.085	cation channel activity	*CACNA1B, CHRNA9, CHRNB4, LRRC55, SNAP25*
6.41E-03	0.017	sodium:sulfate symporter activity	*SLC13A1*
Transmembrane transporter activity			

4.66E-05	0.068	postsynaptic neurotransmitter receptor activity	*CHRNA9*, *CHRNB4*, *DRD3*, *GABRG2*
2.26E-04	0.051	neuropeptide receptor binding	*CRH*, *GAL*, *MRAP*
3.80E-04	0.051	transmitter-gated ion channel activity involved in regulation of postsynaptic membrane potential	*CHRNA9*, *CHRNB4*, *GABRG2*
4.06E-04	0.051	neuropeptide receptor activity	*GAL*, *PRLHR*, *SSTR1*
4.45E-04	0.068	neurotransmitter receptor activity	*CHRNA9*, *CHRNB4*, *DRD3*, *GABRG2*
4.62E-04	0.051	neurotransmitter receptor activity involved in regulation of postsynaptic membrane potential	*CHRNA9*, *CHRNB4*, *GABRG2*
7.59E-04	0.102	gated channel activity	*CACNA1B*, *CHRNA9*, *CHRNB4*, *GABRG2*, *LRRC55*, *SNAP25*
9.00E-04	0.034	anion:sodium symporter activity	*SLC13A1*, *SLC18A2*
9.46E-04	0.051	transmitter-gated ion channel activity	*CHRNA9*, *CHRNB4*, *GABRG2*
9.46E-04	0.051	transmitter-gated channel activity	*CHRNA9*, *CHRNB4*, *GABRG2*
1.18E-03	0.034	acetylcholine-gated cation-selective channel activity	*CHRNA9*, *CHRNB4*
1.67E-03	0.051	extracellular ligand-gated ion channel activity	*CHRNA9*, *CHRNB4*, *GABRG2*
2.25E-03	0.034	anion:cation symporter activity	*SLC13A1*, *SLC18A2*
2.25E-03	0.034	syntaxin-1 binding	*SNAP25*, *SYT4*
2.25E-03	0.034	GTP-dependent protein binding	*GCH1*, *RAB3C*
2.51E-03	0.102	metal ion transmembrane transporter activity	*CACNA1B*, *CHRNA9*, *LRRC55*, *SLC13A1*, *SLC18A2*, *SNAP25*
2.57E-03	0.102	ion channel activity	*CACNA1B*, *CHRNA9*, *CHRNB4*, *GABRG2*, *LRRC55*, *SNAP25*
3.89E-03	0.034	excitatory extracellular ligand-gated ion channel activity	*CHRNA9*, *CHRNB4*
4.36E-03	0.102	passive transmembrane transporter activity	*CACNA1B*, *CHRNA9*, *CHRNB4*, *GABRG2*, *LRRC55*, *SNAP25*

6.41E-03	0.017	peptidyl-dipeptidase activity	*ACE2*
Other			
3.21E-03	0.017	GTP cyclohydrolase activity	*GCH1*
3.21E-03	0.017	GTP cyclohydrolase I activity	*GCH1*
3.21E-03	0.017	galanin receptor binding	*GAL*
3.21E-03	0.017	type 1 galanin receptor binding	*GAL*
3.21E-03	0.017	type 2 galanin receptor binding	*GAL*
3.21E-03	0.017	type 3 galanin receptor binding	*GAL*
